# Percentages of CD4+CD8+ Double-positive T Lymphocytes in the Peripheral Blood of Adults from a Blood Bank in Bogotá, Colombia

**DOI:** 10.4274/tjh.galenos.2019.2019.0256

**Published:** 2020-02-20

**Authors:** Miguel S. Gonzalez-Mancera, Natalia I. Bolaños, Manuel Salamanca, Guillermo A. Orjuela, Ayda N. Rodriguez, John M. Gonzalez

**Affiliations:** 1 University of los Andes, School of Medicine, Grupo de Ciencias Básicas Médicas, Bogotá, Colombia; 2 National Blood Bank, Colombian Red Cross, Bogotá, Colombia

**Keywords:** Flow cytometry, Lymphocyte, Lymphocyte subpopulation, T lymphocytes

## Abstract

**Objective:**

CD4+CD8+ double-positive T-cells (DPTs) have been classified as a separate T-cell subpopulation, with two main phenotypes: CD4^high^ CD8^low^ and CD4^low^ CD8^high^. In recent years, the relevance of DPTs in the pathogenesis of infections, tumors, and autoimmune diseases has been recognized. Reference values among healthy individuals remain unknown. Therefore, the aim of this study is to provide a reference value for DPTs in peripheral blood from healthy donors in a blood bank in Bogotá, Colombia, and to determine the activation status using a surface marker.

**Materials and Methods:**

One hundred healthy donors were enrolled in the study. Peripheral blood cells were stained for CD3, CD4, CD8, and CD154 (CD40L), and cellular viability was assessed with 7-aminoactinomycin D and analyzed by flow cytometry.

**Results:**

The median value for DPTs was 2.6% (interquartile range=1.70%-3.67%). Women had higher percentages of DPTs than men (3.3% vs. 2.1%). The subpopulation of CD4^low^ CD8^high^ showed higher expression of CD154 than the other T-cell subpopulations.

**Conclusion:**

DPT reference values were obtained from blood bank donors. A sex difference was found, and the CD4^low^ CD8^high^ subpopulation had the highest activation marker expression.

## Abstract (tr)

**Amaç:**

CD4+CD8+ çift pozitif T hücreleri (ÇPT) ayrı bir T hücre alt popülasyonu olarak iki temel fenotip ile sınıflandırılmaktadır: CD4^yüksek^ CD8^düşük^ ve CD4^düşük^ CD8^yüksek^. Son yıllarda, ÇPT’lerin enfeksiyonlar, tümörler ve otoimmün hastalıkların patogenezi ile ilişkisi tanımlanmıştır. Sağlıklı bireyler arasında referans değerleri bilinmemektedir. Bu nedenle, bu çalışmanın amacı, Kolombiya Bogota’daki bir kan bankasındaki sağlıklı vericilerden alınan periferik kandaki ÇPT’ler için bir referans değeri sağlamak ve bir yüzey belirteci kullanarak aktivasyon durumunu belirlemektir.

**Gereç ve Yöntemler:**

Çalışmaya yüz sağlıklı verici dahil edilmiştir. Periferik kan hücreleri CD3, CD4, CD8 ve CD154 (CD40L) için işaretlendi ve hücresel canlılık 7-aminoactinomycin D kullanarak akan hücre ölçer ile analiz edildi.

**Bulgular:**

ÇPT’ler için ortanca değer %2,6 (çeyrekler arası aralık=%1,70-%3,67) olarak saptandı. Kadınlarda ÇPT yüzdesi erkeklere göre daha yüksek bulundu (%3,3 karşı %2,1). CD4^düşük^ CD8^yüksek^ alt popülasyonu diğer T hücre alt popülasyonlarından daha yüksek CD154 ekspresyonu gösterdi.

**Sonuç:**

ÇPT referans değerleri kan bankası vericilerinden elde edilmiştir. Cinsiyetler arası fark ve CD4^düşük^ CD8^yüksek^ alt popülasyonunda da en yüksek aktivasyon belirteci ekspresyonu saptanmıştır.

## Introduction

Classically, T-cells have been classified according to the cell surface markers CD4 and CD8. The expression of these proteins is considered to be a mutually exclusive event reflecting the specific functions of each major T-cell population in peripheral blood: CD4+ or helper T-cells and CD8+ or cytotoxic T-cells. However, with the use of multiparametric cellular analysis methods, a variety of minor T-cell subpopulations have been described [1], such as mature CD4+CD8+ or double-positive T-cells (DPTs) [2,3]. This T-cell phenotype was initially described in the thymus, where more than 80% of thymocytes expressed both CD4+CD8+, which later commit to one cell lineage (CD8+ or CD4+) after interaction with human leukocyte antigen (HLA) class I or II molecules, respectively [4]. The origin of DPTs in the peripheral blood of healthy individuals has been attributed to the premature release of CD4+CD8+ T-cells from the thymus to the periphery [5,6,7]. However, additional studies have suggested that DPTs could originate from the acquisition of the second marker by single-positive (either CD4+ or CD8+) T-cells in the periphery [6,8]. Although several investigations support that mature CD4+ T-cells are the source of DPTs, there is also evidence that CD8+ T-cells could be the primary cellular type [6]. Unlike immature thymic DPTs, peripheral DPTs exhibit the functional properties of mature T-cells, including antigen-dependent cytokine production, cytolytic activity, and expression of markers associated with the memory phenotype [9,10]. DPTs are divided into two main populations based on the differential expression of each marker (CD4^high ^CD8^low^ and CD4^low ^CD8^high^) [1,2,3]. In healthy donors, CD4^high ^CD8^low^ cells have an effector or memory phenotype (T_EM_), whereas CD4^low ^CD8^high^ cells display a central memory phenotype (T_CM_), which can switch to an effector phenotype during viral infections such as hepatitis C virus (HCV) and human immunodeficiency virus (HIV) [9,10]. Little is known about the functionality of DPTs, though their function seems to be disease-specific. DPTs exhibit cytotoxic potential in chronic viral infections, such as lymphocytic choriomeningitis virus [11] and HIV [12], and in certain types of cancer [13,14,15]. DPTs can have a regulatory role in malignancies [13,14], systemic sclerosis [16], and inflammatory bowel disease [17]. In autoimmune diseases, DPTs can be found in different compartments; they increase in peripheral blood among patients with myasthenia gravis [18] but are found infiltrating the affected tissues in autoimmune thyroid disease and rheumatoid arthritis [19,20]. In systemic sclerosis and rheumatoid arthritis, DPTs secrete mainly interleukin-4 [16,19], whereas in tumors, such as melanoma and cutaneous lymphoma, the primary cytokine produced is tumor necrosis factor (TNF)-α [13,14]. In chronic parasitic infections such as Chagas disease, DPTs are not only increased in peripheral blood [21] but are also found infiltrating the cardiac tissue in patients with advanced chagasic cardiomyopathy [22,23].

Due to the growing interest in the study of DPT subpopulations and their potential roles in specific diseases, it seems essential to determine reference values among healthy individuals. Therefore, the main goal of this study is to establish standard values of DPTs and to evaluate their functional profile by determining the presence of one specific activation marker in suitable donors from a blood bank in Bogotá, Colombia.

## Materials and Methods

### Study and Donors

This is a descriptive and cross-sectional study of suitable donors who volunteered for blood donation in 2017 at the National Blood Bank of the Colombian Red Cross in Bogotá, Colombia. The protocol and informed consent was approved by the Ethical Committee of the University of los Andes (Act 209 of 2013). One hundred and three donors were enrolled in this study and provided informed consent. The demographic characteristics of our study population are shown in Table 1. Three individuals were excluded due to reactive serological tests for syphilis. The study population included 55 men and 45 women who fulfilled the donation requirements and had negative screening tests (HIV, syphilis, hepatitis C virus, hepatitis B virus, Chagas disease, and human T-cell lymphotropic virus). They ranged from 19 to 61 years of age. Samples were obtained from citrate phosphate dextrose anticoagulated blood bags and transported refrigerated from the blood bank to the biomedical sciences laboratory where the cellular analyses were conducted.

### Cell Labeling and Cytometry Acquisition

**Figure 1:**
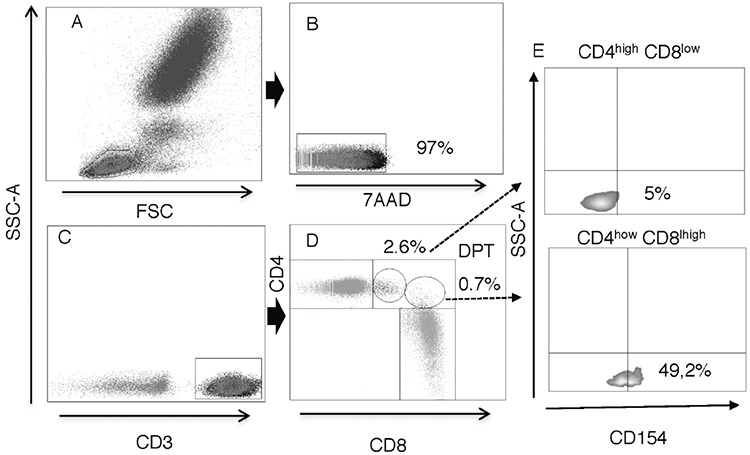
Flow cytometry gating strategy. A) Peripheral blood leukocytes cells distributed in dot plot by flow cytometry according to FSC versus SSC. B) Cell viability using 7-aminoactinomycin D. C) T-cells identified by the expression of CD3. D) Dot plot distribution showing the expression of CD4 and CD8, and the gate on DPTs: CD4^high^ CD8^low^ and CD4^low^ CD8^high^. E) Density plot showing CD154 expression in each DPT subpopulation. SSC: Side scatter, FSC: Forward scatter, DPTs: Double-positive T-cells, DPT: Double-positive T-cell.

Blood samples of 100 µL were used for labeling. Antibodies included anti-CD3 APC (clone UCHT1), anti-CD4 PerCP (SK3), anti-CD8 FITC (SK1), and anti-CD154 PE (TRAP1). All antibodies were purchased from BD Pharmingen (BD, San Diego, CA, USA). Blood was stained in darkness for 30 min at 4°C and then incubated with a cell lysis buffer (BD FACS Lysing Solution) for 15 min at room temperature. Subsequently, cells were washed twice in phosphate-buffered saline (PBS) (Sigma-Aldrich, St. Louis, MO, USA) (0.01 M, pH 7.4, PBS 1X) and gently resuspended. Viability was assessed with 7-aminoactinomycin D staining (7-AAD, BD). Samples were acquired and analyzed with a FACSCanto II flow cytometer with FACSDiva 6.1 software (BD Bioscience, San Jose, CA, USA). At least 5x10^4^ cells were acquired in the CD3+ T lymphocyte population gate according to their forward scatter (FSC) and side scatter (SSC) features. The gating strategy for CD3+ T-cells and DPTs is shown in Figure 1.

### Statistical Analysis

Information about the donor characteristics is given in percentages. The Shapiro-Wilk normality test was conducted for all data obtained from the cellular analysis. A nonparametric statistical analysis was performed in the study. The Mann-Whitney U test was used to compare between two groups. The Kruskal-Wallis test was used to compare among multiple groups. The results are shown as medians and interquartile ranges (IQRs). Statistical analysis was performed using GraphPad Prism 7 software (San Diego, CA, USA). Significance was established at p<0.05.

## Results

**Figure 2:**
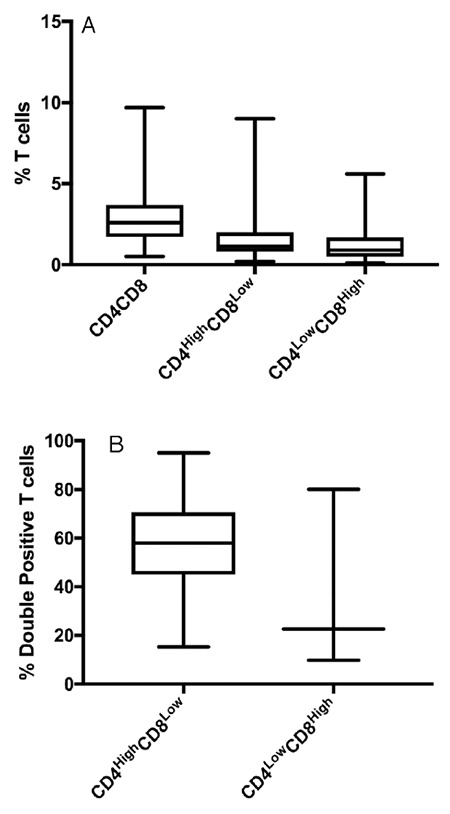
A) Percentage of subpopulations of DPTs from the total T lymphocytes. B) Percentage of subpopulations among the total DPTs. Data are displayed as medians with minimums and maximums. DPTs: Double-positive T-cells.

**Figure 3:**
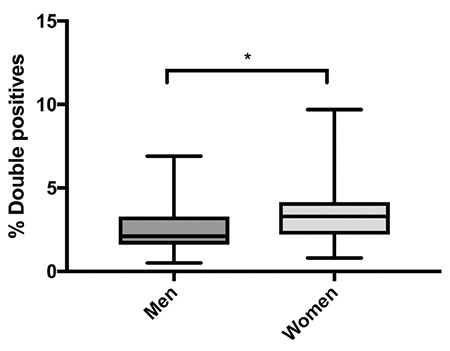
Percentage of the total DPTs according to sex. Women had higher percentages of DPT cells than men. Mann-Whitney, p=0.007. Data are displayed as medians with minimums and maximums. DPT: Double-positive T-cell.

**Figure 4:**
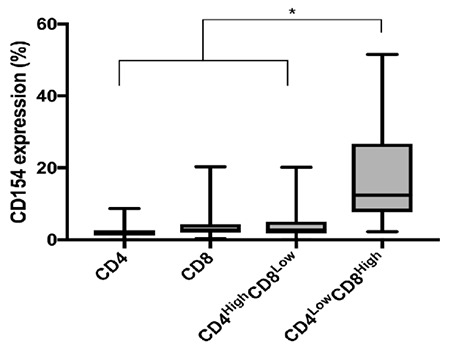
Expression of CD154 in single-positive and double-positive T-cell subpopulations. The subpopulation CD4^low^ CD8^high^ had higher CD154 expression than other subpopulations of T-cells. Data are displayed as medians with minimums and maximums.

T lymphocytes are a highly heterogeneous group of immune cells that have been of great interest in clinical and biomedical research studies. In an effort to establish values between the different subpopulations of DPTs, lymphocytes were analyzed from peripheral blood mononuclear cells from each blood donor included in the study. Lymphocytes were gated according to FSC vs. SSC features, as shown in Figure 1A. The median cell viability was 99.15% (IQR=98.8%-99.4%) in all samples, as shown in Figure 1B. CD3+ T-cells were subsequently identified according to cell surface expression of CD4 or CD8, as shown in Figures 1C and 1D, respectively. DPTs were classified into two main subpopulations, CD4^high ^CD8^low^ and CD4^low ^CD8^high^, as shown in Figure 1D. The median total percentage of DPTs among CD3+ T-cells in all samples studied was 2.6% (IQR=1.7%-3.67%), and the CD4^high ^CD8^low^ subpopulation showed a median content of 1.15% (IQR=0.8%-2.0%), whereas in the CD4^low ^CD8^high^ subpopulation, it was 0.9% (IQR=0.5%-1.67%), as shown in Figure 2A. CD4^high ^CD8^low^ accounted for 57.97% of DPTs, as shown in Figure 2B. Total DPTs were analyzed according to sex. Women showed a higher percentage of DPTs (median=3.3%; IQR=2.2%-4.15%) than men (median=2.1%; IQR=1.6%-3.3%), p=0.007, as shown in Figure 3. The activation status of DPTs was assessed by using the surface marker expression of CD154, also called CD40L, as shown in Figure 1E. The subpopulation of CD4^low ^CD8^high^ showed higher expression of CD154 than the other T-cell populations (p≤0.0001), as shown in Figure 4*.*

## Discussion

In recent decades, there has been growing interest in CD4+CD8+ double-positive T lymphocytes, which are considered a separate subpopulation of T-cells associated with different pathologic conditions. In this study, a reference percentage value was established among DPT subpopulations. An activation marker was also studied in the blood samples of volunteer blood bank donors. An increased frequency of DPTs was found in women compared to men. Sex variance has been found in other blood cell subpopulations, such as natural killer lymphocytes [24,25], and it would be of particular interest to evaluate DPTs during pregnancy and in placental tissue due to the sex difference found.

The frequency of DPTs in peripheral blood does not increase in HIV, HCV, or melanoma; however, this subpopulation exhibited a higher expression of surface activation markers (i.e. HLA-DR and CD38) and greater cytokine production (i.e. interferon-γ and TNF-α) in individuals with these diseases when compared to controls [7,9,10,14]. Nonetheless, in one study assessing HIV, an increased frequency of DPTs expressing CD38 and HLA-DR was associated with advanced disease in patients with CD4+ counts of <200 cells/µL [7], which are markers that have been widely used to define T-cell activation by antigens [26]. Additionally, an increased frequency of DPTs in peripheral blood was found in chronic chagasic patients [22,23] and among individuals with myasthenia gravis [18], and the percentage of DPTs interestingly decreased after treatment in both diseases [18,23]. Among patients with melanoma, there was an increased frequency of DPTs in draining lymphoid nodes and tumor-infiltrating lymphocytes [14].

In this study, a higher expression of CD154 (CD40L) was found in CD4^low ^CD8^high ^cells. This activation marker has been used as an indicator for antigen-specific T-cell activation in CD4+ T-cells [27] and in CD8+ T-cells [28]. Remarkably, this DPT subpopulation has an effector memory phenotype [9,10]. CD154 is the ligand of CD40, and this axis has been found to be of particular interest in the therapy of autoimmune diseases [29]. It would be very important to elucidate the functional role of CD154 in DPTs. Other markers have been studied on DPT cells, including activation, homing, and differentiation markers [10,22]. Due to the role of DPTs in the pathogenesis of several diseases, it seems promising to study the expression of inhibitory molecules such as PD-1 or CTLA-4. These molecules are currently targets of immunotherapy for different tumor conditions [30,31,32]. In previous reports, no other activation markers, such as CD38 or HLA-DR, were found in DPTs from healthy donors [7,10,22].

In this study, a median DPT rate of 2.6% was found, which was a higher result than that of the control donors in previous studies. For instance, in controls used to study DPTs in HIV patients, the median was 0.8% (IQR=0.1%-1.2%) [7]; in melanoma, the mean was 0.9% [standard deviation (SD) ±0.6] [14]; in HCV infection, the mean was 1% (SD ±0.6) [10]; and in chronic Chagas disease, the mean was 1.1% (±0.5) [22]. However, the control donors analyzed in these studies were from small cohorts, and demographic information about blood donor characteristics was lacking. Our study sample was significantly larger than those included in prior investigations, which could explain the increments evidenced in the results. Indeed, in one study, the frequency of DPTs ranged from 0% to 5% in control donors [18].

## Conclusion

These findings and the differences found between the sexes can be used for future reference in specific populations and diseases. The limitations of this study include the age restriction of our sample and the limited screening tests performed for each donor. To the best of our knowledge, this is the first study to assess the frequency of DPTs in a large cohort of blood bank donors.

## Figures and Tables

**Table 1:**
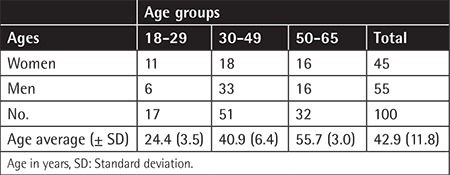
Characteristics of the population studied.
